# Gene Pair Correlation Coefficients in Sphingolipid Metabolic Pathway as a Potential Prognostic Biomarker for Breast Cancer

**DOI:** 10.3390/cancers12071747

**Published:** 2020-07-01

**Authors:** Meena Kishore Sakharkar, Sarinder Kaur Dhillon, Saravana Babu Chidambaram, Musthafa Mohamed Essa, Jian Yang

**Affiliations:** 1 Drug Discovery and Development Research Group, College of Pharmacy and Nutrition, University of Saskatchewan, 107 Wiggins Road, Saskatoon, SK S7N 5E5, Canada; 2 Institute of Biological Sciences, Faculty of Science, University of Malaya, Kuala Lumpur 50603, Malaysia; sarinder@um.edu.my; 3 Department of Pharmacology, JSS College of Pharmacy, JSS Academy of Higher Education & Research (JSSAHER), Mysuru 570 015, India; babupublications@gmail.com; 4 Ageing and Dementia Research Group, Sultan Qaboos University, Muscat 123, Oman; drmdessa@gmail.com; 5 Department of Food Science and Nutrition, College of Agricultural and Marine Sciences, Sultan Qaboos University, Muscat 123, Oman

**Keywords:** sphingolipid, gene expression, gene pair correlation, breast cancer, biomarker

## Abstract

Complex diseases such as cancer are usually governed by dynamic and simultaneous modifications of multiple genes. Since sphingolipids are potent bioactive molecules and regulate many important pathophysiological processes such as carcinogenesis, we studied the gene pair correlations of 36 genes (31 genes in the sphingolipid metabolic pathway and 5 genes encoding the sphingosine-1-phosphate receptors) between breast cancer patients and healthy controls. It is remarkable to observe that the gene expressions were widely and strongly correlated in healthy controls but in general lost in breast cancer patients. This study suggests that gene pair correlation coefficients could be applied as a systematic and novel method for the diagnosis and prognosis of breast cancer.

## 1. Introduction

Breast cancer is the most prevalent type of cancer in women with almost 2.1 million new cases and 627 thousand deaths worldwide in 2018 [1]. Increased mammography screening has played a critical role in the significant decrease in the age-standardized mortality rate (ASMR), alongside the use of more efficient therapies such as targeted therapy [2,3,4]. Although ASMR has dropped from 42.7 deaths per 100,000 in 1986 to a projected 22.4 deaths per 100,000 in 2019 in Canada [2], developing more effective early diagnosis technologies is crucial for breast cancer patients’ survival.

**Figure 1:**
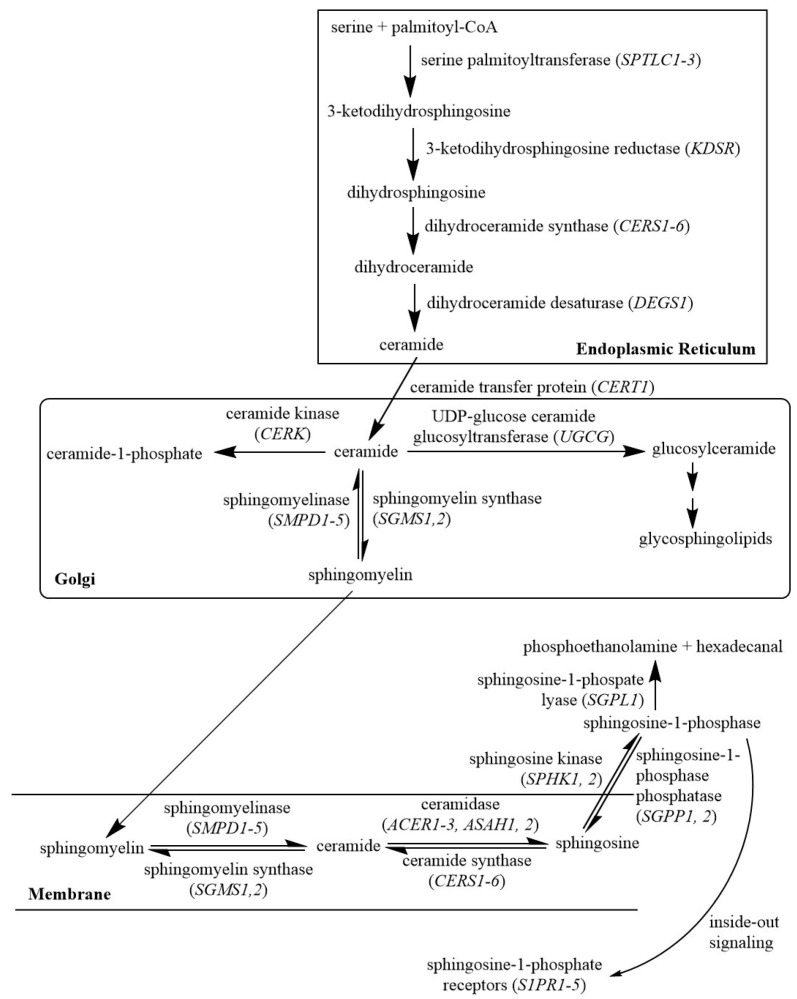
Sphingolipid metabolic pathway (plus five sphingosine-1-phosphate receptors).

Sphingolipids act as potent bioactive molecules and play critical roles in a broad spectrum of physiological and pathophysiological processes, specifically cancer [5,6,7,8]. For example, ceramide inhibits growth and induces apoptosis of cancer cells, whereas sphingosine-1-phosphate (S1P) promotes proliferation and survival of cancer cells [9,10]. Interestingly, the sphingolipid metabolic pathway, shown in Figure 1, contains a very limited number of members and is highly conserved among eukaryotes. The metabolism and functions of sphingolipids in cancer are far from completely understood despite a vast amount of research during the past twenty years. Several studies have investigated bioactive sphingolipids as biomarkers of tumorigenesis [11,12], however, most of these biomarker investigations reported only changes in gene expression using microarray or RNASeq arrays. Since complex diseases such as cancer are dynamically governed by a combination of multiple genes, it would be prudent to monitor alterations in gene correlations as a measure of disease progression. In agreement with this, Magen et al. recently reported that pairwise gene expression states are associated with cancer patient survival [13]. Our previous study has also suggested that gene expression correlation coefficients could be used for cancer diagnosis and prognosis [14].

## 2. Results and Discussion

**Figure 2:**
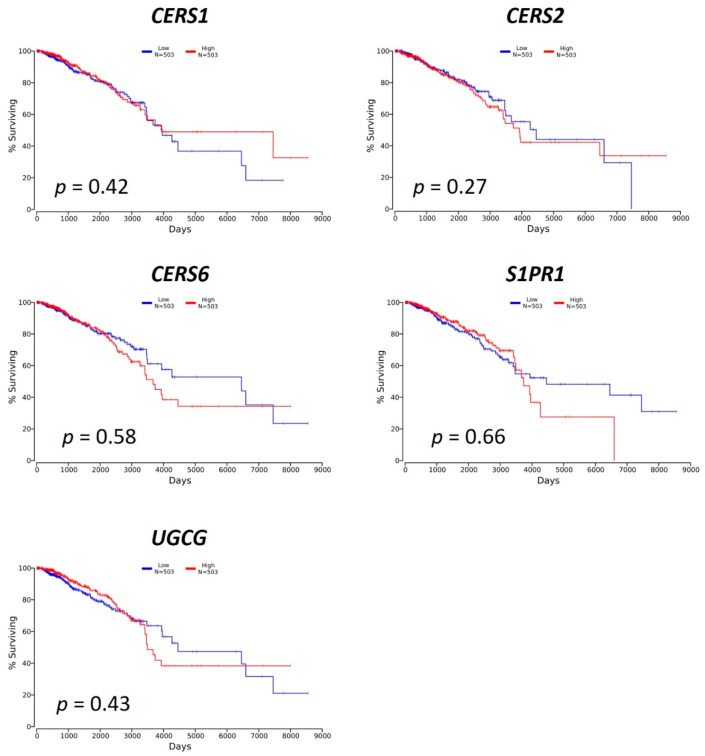
Kaplan–Meier plots of the patient survival probability versus the mRNA expression level of *CERS1*, *CERS2*, *CERS6*, *S1PR1* and *UGCG* based on the patients’ data TCGA-BRCA. The plots were generated using OncoLnc [19] with low and high expressions shown in blue (*n* = 503) and red (*n* = 503), respectively. None of the genes were identified as being a prognostic factor for breast cancer (*p* < 0.05).

We extracted the gene expression information for 36 genes, including 31 genes in the sphingolipid metabolic pathway and 5 genes encoding S1P receptors (S1PRs), from The Cancer Genome Atlas (TCGA) dataset TCGA-BRCA, which contains RNASeq expression data and matching clinical samples from 1102 breast cancer patients and 113 healthy controls. Only 6 genes were up- or down-regulated by more than 2-fold (*p* < 0.05) out of the 36 genes, shown in Table 1. The up-regulated genes were *CERS1* (ceramide synthase 1, log_2_ = 1.16), *CERS2* (ceramide synthase 2, log_2_ = 1.30), *CERS6* (ceramide synthase 6, log_2_ = 1.06), *SMPD5* (sphingomyelin phosphodiesterase 5, pseudogene, log_2_ = 1.66) and *UGCG* (UDP-glucose ceramide glucosyltransferase, log_2_ = 1.22), whereas the down-regulated gene was *S1PR1* (sphingosine-1-phosphate receptor 1, log_2_ = −1.93). Alternations of expression of *CERS2*, *CERS6*, *S1PR1*, *SMPD5* and *UGCG* are consistent with previous studies [15,16,17,18], however, the expression of *CERS1* was determined to be very low in breast cancer [17]. To further investigate the effects of these genes (except pseudogene *SMPD5*) on breast cancer patient survival, we calculated the Kaplan–Meier plot for each of the genes using OncoLnc [19]. As shown in Figure 2, none of these genes were identified to be a prognostic factor for human breast invasive carcinoma although they might be potential diagnostic factors. 

**Figure 3:**
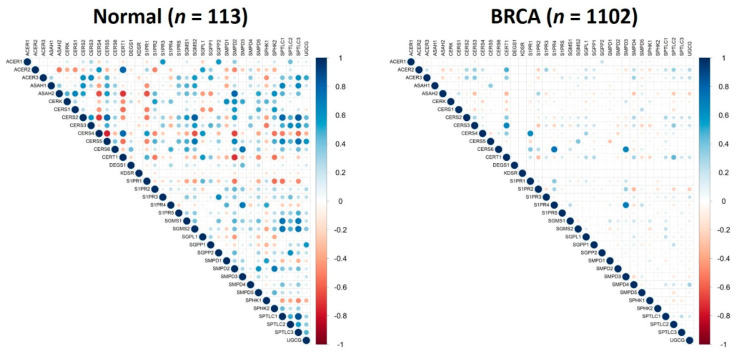
Gene pair correlations of 36 sphingolipid-related genes in healthy controls (*n* = 113) and breast cancer patients (*n* = 1102). Positive and negative correlations are represented by blue and red dots, respectively, and the sizes of the dots are proportional to the correlation values. The 36 sphingolipid-related genes contain 31 genes in the sphingolipid metabolic pathway and 5 genes encoding the sphingosine-1-phosphate receptors (S1PRs).

**Figure 4:**
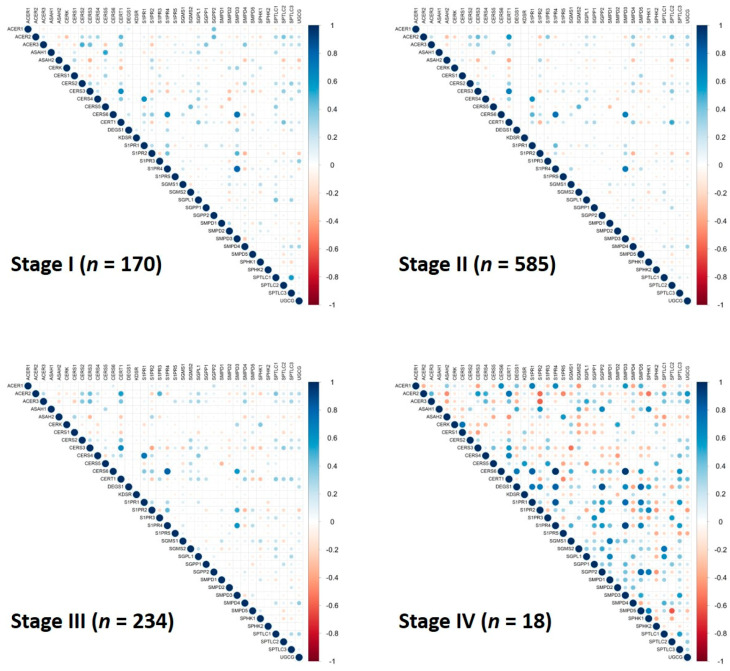
Gene pair correlations of 36 sphingolipid-related genes in stage I breast cancer patients (*n* = 170), stage II breast cancer patients (*n* = 585), stage III breast cancer patients (*n* = 234) and stage IV breast cancer patients (*n* = 18). Positive and negative correlations are represented by blue and red dots, respectively, and the sizes of the dots are proportional to the correlation values. The 36 sphingolipid-related genes contain 31 genes in the sphingolipid metabolic pathway and 5 genes encoding the sphingosine-1-phosphate receptors (S1PRs).

Human physiological and pathophysiological processes commonly employ a coordination of multiple genes. It is reasonable to assume that genes would decouple (loss of correlation) or couple (gain of correlation) prior to changing their expression levels during an alternation of a biological process such as carcinogenesis. Although previous studies reported that certain genes became pair-correlated during cancer progression [13,14,20,21], gene pair correlation coefficient or pattern change in gene pair correlations of a group of genes has never been proposed as a diagnostic or prognostic factor for any type of cancer. In the current study, we evaluated the significance of gene expression correlation coefficients and pattern change in gene pair correlations for the 36 sphingolipid-related genes in breast invasive carcinoma. Pairwise correlation coefficients were calculated for both healthy control and breast cancer patient data. As illustrated in Figure 3, the genes were widely and strongly correlated in healthy controls. Surprisingly, these correlations were in general lost in breast cancer patients with only five pairs of genes (*CERS3*-*CERT1*, *CERS4*-*S1PR1*, *CERS6*-*S1PR4*, *CERS6*-*SMPD3*, and *S1PR4*-*SMPD3*) remaining in medium/strong positive-correlations. Interestingly, genes *CERS3* and *CERT1* were not correlated in healthy controls but became positively correlated in breast cancer patients. We further analyzed the pairwise gene correlations in the different stages of breast cancer, as seen in Figure 4, to gain insight into the stage of disease progression at which these gene pair correlations disappear in comparison to healthy controls. It is remarkable to observe that gene pair correlation coefficients for the 36 sphingolipid-related genes were gradually reduced up to stage III of the cancer and then increased in values and “regain and revive” in stage IV. These data imply that the sphingolipid metabolic pathway reclines its member coordination in order to alleviate its potent biological functions and allow faster growth of breast cancer cells. Upon local or distal metastasis, the pathway recuperates its member coordination and critical functions to ensure settlement of breast cancer cells in a new tissue/organ environment. The current observation of losing gene pair correlations for the sphingolipid metabolic pathway and S1PRs in breast cancer suggests two potential applications. First, follow-up of the gene pair correlation coefficients for the sphingolipid metabolic pathway and S1PRs could be a potentially valid approach for early detection of breast cancer. Secondly, population genomic screening of these sphingolipid-related genes could provide a glimpse of breast cancer distribution and even stage information within a community. Because of the very limited number of patients with disease stage information (stage I: 170, stage II: 585, stage III: 234, and stage IV: 18), especially for stage IV, further studies are required to confirm the trend of gene pair correlations during breast cancer progression. Nonetheless, this study clearly supports the important role of the sphingolipid pathway in breast cancer and offers a systematic and novel method to use gene pair correlation coefficients and/or pattern change in gene pair correlation coefficients for diagnosis and prognosis of breast cancer. We are currently developing a mathematical algorithm to quantitatively compare the pattern changes of gene pair correlation coefficients and will validate this algorithm as a potential diagnosis and prognosis of breast cancer using patients’ tissue samples.

## 3. Materials and Methods

### 3.1. Data Acquisition

RNAseq data set (TCGA-BRCA) was extracted from The Cancer Genome Atlas (TCGA) via the Genomic Data Commons (GDC) data portal [22]. Using GDC Data Transfer Tool, we downloaded 1092 breast cancer cases. A total of 1215 samples (1102 tumor and 113 normal) were obtained from the cases. Sample sheet file was also downloaded to extract the clinical information for the samples. We obtained 170 stage I, 585 stage II, 234 stage III, and 18 stage IV samples. For every subject, we analyzed the expression of 60,483 RNA transcripts in terms of FPKM values.

### 3.2. Identification and Visualization of Differentially Expressed Genes

Differentially expressed genes (DEGs) in tumor against normal and among the stages of tumor were identified using the DEGseq package from R [23]. Likelihood Ratio Test (LRT) was applied, and the sample expression profiles were screened using *p*-value < 0.001. The output was expressed in normalized log_2_ fold-change (Log_2_FC). We then manually extracted the information on expression changes of 36 sphingolipid-related genes (31 sphingolipid metabolic pathway and 5 S1P receptors) out of 1102 tumor patient samples using normal healthy individual samples (*n* = 113) as controls.

### 3.3. Computation of Correlation Matrix

Correlation matrix was computed for each of the subgroups using the cor function in R. Visualization of the matrix was done using the corrplot function in R. Positive and negative correlations are represented by blue and red dots, respectively, and the sizes of the dots are proportional to the correlation values.

## 4. Conclusions

Breast cancer is the most common type of cancer in women. Despite recent advances in diagnosis and treatment, prognosis for late-stage and recurrent breast cancer patients remains poor. Early diagnosis is still essential for patient’s survival. In this study, the functional role of the sphingolipid metabolic pathway, which is highly conserved in eukaryotes, was investigated in human breast cancer. Using the TCGA-BRCA dataset, we showed that five genes (*CERS1*, *CERS2*, *CERS6*, *UGCG* and pseudogene *SPMD5*) were upregulated more than two-fold and one gene (*S1PR1*) was downregulated more than two-fold. However, none of these genes were identified as being a prognostic factor for human breast invasive carcinoma. We also calculated gene pair correlation coefficients for the 36 sphingolipid-related genes. These genes were widely and strongly correlated in healthy controls but lost their correlations in breast cancer patients. Specifically, gene pair correlation coefficients were gradually reduced up to stage III and then increased in values and “regain and revive” in stage IV. This suggests that gene pair correlation coefficient and/or pattern change in gene pair correlation coefficients for a group of genes could be applied as potential diagnostic and/or prognostic biomarkers for human breast cancer.

## Figures and Tables

**Table 1:** Expression changes (normalized log_2_FC) of 36 sphingolipid-related genes between healthy controls (*n* = 113) and breast cancer patients (*n* = 1102). Genes up- and down-regulated by more than 2-fold (i.e., |log_2_FC| ≥ 1) were highlighted in red and blue, respectively. The selected 36 genes contain 31 genes in the sphingolipid metabolic pathway and 5 genes encoding the sphingosine-1-phosphate receptors (S1PRs).

Gene Name	Log_2_FC	*p* Value	Gene Name	Log_2_FC	*p* Value
*ACER1*	−0.05	0.75	*S1PR4*	0.69	<0.01
*ACER2*	−0.68	<0.01	*S1PR5*	0.51	<0.01
*ACER3*	−0.27	<0.01	*SGMS1*	−0.14	0.02
*ASAH1*	0.13	<0.01	*SGMS2*	−0.35	<0.01
*ASAH2*	0.25	0.69	*SGPL1*	0.72	<0.01
*CERK*	−0.13	<0.01	*SGPP1*	−0.31	<0.01
*CERS1*	1.16	<0.01	*SGPP2*	0.23	0.02
*CERS2*	1.30	<0.01	*SMPD1*	0.14	<0.01
*CERS3*	−0.04	0.83	*SMPD2*	0.79	<0.01
*CERS4*	0.87	<0.01	*SMPD3*	0.90	<0.01
*CERS5*	0.23	<0.01	*SMPD4*	0.62	<0.01
*CERS6*	1.06	<0.01	*SMPD5*	1.66	<0.01
*CERT1*	−0.85	<0.01	*SPHK1*	0.67	<0.01
*DEGS1*	0.45	<0.01	*SPHK2*	0.51	<0.01
*KDSR*	−0.44	<0.01	*SPTLC1*	−0.03	0.44
*S1PR1*	−1.93	<0.01	*SPTLC2*	0.28	<0.01
*S1PR2*	−0.32	<0.01	*SPTLC3*	−0.28	<0.01
*S1PR3*	0.49	<0.01	*UGCG*	1.22	<0.01

## References

[1] Bray F., Ferlay J., Soerjomataram I., Siegel R.L., Torre L.A., Jemal A. (2018). Global cancer statistics 2018: GLOBOCAN estimates of incidence and mortality worldwide for 36 cancers in 185 countries. CA Cancer J. Clin..

[2] Canadian Cancer Statistics Advisory Committee (2019). Canadian Cancer Statistics 2019.

[3] Shields M., Wilkins K. (2009). An update on mammography use in Canada. Health Rep..

[4] Holford T.R., Cronin K.A., Marriotto A.B., Feuer E.J. (2006). Changing patterns in breast cancer incidence trends. J. Natl. Cancer Inst. Monogr..

[5] Maceyka M., Spiegel S. (2014). Sphingolipid metabolites in inflammatory disease. Nature.

[6] Huang Y., Mao K., Chen X., Sun M.A., Kawabe T., Li W., Usher N., Zhu J., Urban J.F., Paul W.E. (2018). S1P-dependent interorgan trafficking of group 2 innate lymphoid cells supports host defense. Science.

[7] Goyal G., Zheng J., Adam E., Steffes G., Jain M., Klavins K., Hummel T. (2019). Sphingolipid-dependent Dscam sorting regulates axon segregation. Nat. Commun..

[8] Hammerschmidt P., Ostkotte D., Nolte H., Gerl M.J., Jais A., Brunner H.L., Sprenger H.G., Awazawa M., Nicholls H.T., Turpin-Nolan S.M. (2019). CerS6-Derived Sphingolipids Interact with Mff and Promote Mitochondrial Fragmentation in Obesity. Cell.

[9] Dadsena S., Bockelmann S., Mina J.G.M., Hassan D.G., Korneev S., Razzera G., Jahn H., Niekamp P., Müller D., Schneider M. (2019). Ceramides bind VDAC2 to trigger mitochondrial apoptosis. Nat. Commun..

[10] Pyne N.J., Pyne S. (2010). Sphingosine 1-phosphate and cancer. Nat. Rev. Cancer.

[11] Saddoughi S.A., Garrett-Mayer E., Chaudhary U., O’Brien P.E., Afrin L.B., Day T.A., Gillespie M.B., Sharma A.K., Wilhoit C.S., Bostick R. (2011). Results of a phase II trial of gemcitabine plus doxorubicin in patients with recurrent head and neck cancers: Serum C_18_-ceramide as a novel biomarker for monitoring response. Clin. Cancer Res..

[12] Knapp P., Bodnar L., Błachnio-Zabielska A., Świderska M., Chabowski A. (2017). Plasma and ovarian tissue sphingolipids profiling in patients with advanced ovarian cancer. Gynecol. Oncol..

[13] Magen A., Das Sahu A., Lee J.S., Sharmin M., Lugo A., Gutkind J.S., Schäffer A.A., Ruppin E., Hannenhalli S. (2019). Beyond Synthetic Lethality: Charting the Landscape of Pairwise Gene Expression States Associated with Survival in Cancer. Cell Rep..

[14] Ling B., Chen L., Liu Q., Yang J. (2014). Gene expression correlation for cancer diagnosis: A pilot study. Biomed. Res. Int..

[15] Abuhussein O., Yang J. (2020). Evaluating the antitumor activity of sphingosine-1-phosphate against human triple-negative breast cancer cells with basal-like morphology. Invest. New Drugs.

[16] Moro K., Kawaguchi T., Tsuchida J., Gabriel E., Qi Q., Yan L., Wakai T., Takabe K., Nagahashi M. (2018). Ceramide species are elevated in human breast cancer and are associated with less aggressiveness. Oncotarget.

[17] Schiffmann S., Sandner J., Birod K., Wobst I., Angioni C., Ruckhäberle E., Kaufmann M., Ackermann H., Lötsch J., Schmidt H. (2009). Ceramide synthases and ceramide levels are increased in breast cancer tissue. Carcinogenesis.

[18] Wegner M.S., Gruber L., Mattjus P., Geisslinger G., Grösch S. (2018). The UDP-glucose ceramide glycosyltransferase (UGCG) and the link to multidrug resistance protein 1 (MDR1). BMC Cancer.

[19] OncoLnc. http://www.oncolnc.org.

[20] Naderi A. (2018). SRARP and HSPB7 are epigenetically regulated gene pairs that function as tumor suppressors and predict clinical outcome in malignancies. Mol. Oncol..

[21] Park B., Lee W., Park I., Han K. (2019). Finding prognostic gene pairs for cancer from patient-specific gene networks. BMC Med. Genom..

[22] TCGA-BRCA. https://portal.gdc.cancer.gov/projects/TCGA-BRCA.

[23] R. https://www.r-project.org.

